# From Acute Injury to Chronic Neurodegeneration: Molecular Mechanisms Linking Secondary Brain Injury to Long-Term Pathology

**DOI:** 10.3390/ijms26157191

**Published:** 2025-07-25

**Authors:** Julia K. Kaniuk, Divy Kumar, Christopher Mazurek, Sepehr Khavari, Christopher Sollenberger, Arun Ahuja, James M. Mossner, Christopher S. Ahuja

**Affiliations:** 1 Feinberg School of Medicine, Northwestern University, 240 E Huron Street, Suite 1-200, Chicago, IL 60611, USA; julia.kaniuk@northwestern.edu (J.K.K.); divy.kumar@northwestern.edu (D.K.); christopher.mazurek@northwestern.edu (C.M.); sepehrkhavari2028@u.northwestern.edu (S.K.); arun.ahuja@northwestern.edu (A.A.); 2 Department of Neurosurgery, The University of Pennsylvania, 3400 Civic Center Blvd, Philadelphia, PA 19104, USA; sollenbc@pennmedicine.upenn.edu; 3 Department of Neurological Surgery, Northwestern Medicine, 676 N St Clair Street, Suite 2210, Chicago, IL 60611, USA; james.mossner@nm.org; 4 Simpson-Querrey Research Institute, 303 E. Superior Street, Chicago, IL 60611, USA

**Keywords:** neurodegenerative disease, cellular and molecular mechanisms, Alzheimer’s Disease (AD), neuroinflammation, traumatic brain injury (TBI), neurotrauma, chronic traumatic encephalopathy, cognitive decline, oxidative stress, mitochondrial dysfunction

## Abstract

Traumatic brain injury (TBI) initiates a complex cascade of pathophysiological events that have far-reaching consequences beyond the initial injury. This review examines the current state of the literature on the mechanisms underlying neurotrauma and neuroinflammation, with particular emphasis on the molecular cross-talk between these disparate pathways that ultimately precipitates the development of chronic traumatic encephalopathy (CTE). We integrate this mechanistic knowledge with potential diagnostic biomarkers, such as glial fibrillary acidic protein (GFAP), neurofilament light chain (NfL), and ubiquitin carboxy-terminal hydrolase L1 (UCH-L1), and advances in neuroimaging and machine learning-based predictive tools. Finally, we discuss the current therapeutic approaches under investigation, and highlight which molecular targets have yet to be explored for potential therapeutic development.

## 1. Introduction

Traumatic brain injury (TBI) is an acquired neurological condition resulting from an external mechanical force that disrupts normal brain function. Rather than being a singular event, TBI initiates a cascade of cellular and molecular events that evolve over time, influencing both short-term recovery and long-term neurological health. TBI is a major cause of death and disability, with over 69,000 TBI-related deaths occurring in the United States in 2021 alone—an average of 190 deaths per day. Falls remain the leading cause of TBI-related hospitalizations, while firearm-related suicide accounts for the most TBI-related deaths [1]. Despite its widespread impact, TBI remains a condition with significant gaps in diagnosis, treatment, and long-term management, particularly due to its evolving and multifaceted pathophysiology.

TBI is typically divided into two distinct phases: primary and secondary injury [2]. The primary injury occurs at the moment of impact and is characterized by direct mechanical damage, including skull fractures, contusions, and diffuse axonal injury [2]. This phase is irreversible, as it results from the initial biomechanical forces exerted on the brain; however, the subsequent secondary injury plays a major role in long-term neurological outcomes and represents a critical target for intervention [3]. Secondary injury develops over hours to weeks and is driven by a complex interplay of molecular and cellular mechanisms, including oxidative stress, excitotoxicity, neuroinflammation, mitochondrial dysfunction, and disruption of cerebral autoregulation [3]. These processes lead to progressive neuronal damage, blood brain barrier (BBB) dysfunction, cerebral edema, and increased intracranial pressure (ICP), all of which contribute to worsening neurological impairment [3].

The continuum from acute injury to chronic neurodegeneration underscores the need for a deeper understanding of the molecular events that bridge these phases. Early disruptions in metabolic homeostasis and inflammatory signaling pathways can predispose the brain to long-term conditions such as chronic traumatic encephalopathy (CTE) and post-traumatic neurodegeneration [4]. Advances in neurocritical care have allowed for more targeted management of secondary brain injury, with intensive care strategies now emphasizing individualized treatment approaches based on patient-specific pathophysiology [4]. Rather than employing a uniform treatment approach, the integration of advanced imaging, cerebral hemodynamic monitoring, and biomarkers has enabled clinicians to tailor interventions aimed at mitigating secondary injury [4]. Understanding the molecular continuum from acute trauma to chronic pathology is essential for developing effective therapeutic strategies that not only improve immediate recovery but also reduce the risk of long-term neurodegenerative consequences. Unlike previous reviews that focus narrowly on specific pathways or clinical endpoints, this review adopts an integrative approach of linking molecular mechanisms to diagnostic biomarkers, imaging, and emerging therapeutics.

## 2. Mechanisms of Secondary Brain Injury

### 2.1. Neuroinflammation

The acute inflammatory response to TBI begins with microglia, the resident macrophages of the CNS. Comprising ~10–12% of the brain, they monitor and mediate inflammation in response to pathogens and cellular damage [5,6]. As such, they can serve both beneficial and deleterious functions in the brain by phagocytosing debris and secreting neurogenic trophic factors while concurrently playing a role in apoptosis [5,7,8,9]. Resting state microglia dynamically survey the CNS, becoming activated by pathogenic stimuli, such as lipopolysaccharide or damage-associated molecular pathogens (DAMPs). Activation shifts microglia morphology, allowing them to travel more easily to the origin of the activating stimulus [5,10].

Microglia are also able to sense other factors resulting from neuronal damage, such as extracellular glutamate, ATP, growth factors, and cytokines, among others [6,11]. A critical component of normal microglial-mediated inflammation is the negative feedback mechanisms that prevent the overactivation of microglia, which can result in further damage to neurons. These negative feedback mechanisms include fractalkine-CX3CR1, CD200–CD200R1, CD47–CD172a/SIRPα, as well as anti-inflammatory mediators such as prostaglandins, neurotrophins, IL-10, and TGF-ß [6,11]. Similarly to peripheral macrophages, microglia demonstrate heterogenous activation into the broad categories of pro-inflammatory M1 or neuroprotective M2 phenotype [12]. While M1 microglia are responsible for phagocytosis and cytokine release, the M2 microglia release antagonistic factors which dampen the inflammatory response, and mediate tissue remodeling post-inflammation [6,12,13].

**Figure 1:**
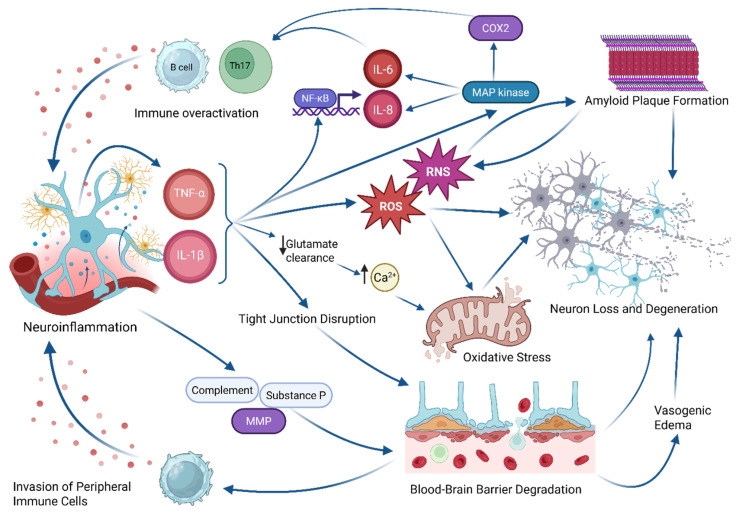
Comprehensive diagram of the neuroinflammatory cascade. Blue arrows indicate progression between stages of the cascade, while black arrows indicate relative changes in the quantity of a substance. Created in BioRender. Ahuja, C. (2025) https://BioRender.com/w7samd7.

The injurious mechanical forces of TBI cause cellular death and BBB disruption, triggering the release of DAMPs [6,14]. Consequently, activated microglia begin the inflammatory process by releasing TNF-α, IL-1ß, reactive oxygen species (ROS), and NO [5,14,15,16,17,18]. In addition to the inflammation brought about by CNS-native immune cells, the disrupted BBB allows peripheral immune cells, like macrophages and neutrophils, to contribute to the inflammatory response, potentially causing acute molecular damage to the neural tissue as pictured in Figure 1 [6,14].

Cytokines, such as TNF-α and IL-1ß, have been heavily implicated in both physiological and pathophysiological states of inflammation. TNF-α has been implicated in p53-mediated neuronal apoptosis and alteration of the BBB permeability through pericyte modulation. IL-1ß, produced by the DAMP-activated protein caspase-1, has been suggested to play a role in increasing lesion size in ischemic brain injury animal models [14,19,20]. Other studies provide evidence that TNF-α and IL-1ß work synergistically to enhance inflammation, possibly through production of NO [21]. TNF-α signaling leads to the upregulation of the apoptotic protein’s Fas-associated death domain (FADD) protein and caspase-8 [22,23]; however, it may also promote cell survival through the activation of the NF-κB signaling pathway, which was shown to inhibit caspase-8 mediated apoptosis [24]. IL-1ß also increases NF-kB activity which, when overactivated, can lead to the persistence of autoreactive B-cells and Th17-cells, fueling neuroinflammation [25]. Additionally, IL-1ß initiates the MAPK pathway which triggers the production of pro-inflammatory cytokines IL-8 and IL-6, and COX2 [22,26]. Both TNF-α and IL-1ß reduce the integrity of the BBB by disrupting tight junctions between endothelial cells, which otherwise exclude peripheral cells and molecules from entering the CNS. Additionally, COX2 activity and amyloid (Aß) plaque development appears to promote migration of peripheral immune cells into the brain, most likely exacerbating neuroinflammation [27,28].

In addition to the cytokine-mediated response, there is evidence suggesting that microglia also exacerbate acute inflammation post-TBI through the release of NO. NO reacts with superoxide produced from NADPH oxidase to form peroxynitrate, a potent inflammatory mediator and cytotoxic molecules [5,29]. Additionally, NO has been implicated in excitotoxicity following TBI, a mechanism that will be discussed later in this review [18]. ROS and NO have also been implicated in the pathogenesis of protein misfolding and may contribute to the formation of Aß plaques following TBI [3,9].

It is a well-documented phenomenon in TBI that acute inflammation immediately following head injury can precipitate chronic inflammation that can last for decades. This cumulative process appears to be correlated with the disruption of the BBB and invasion of peripheral immune cells, though it can be difficult to ascertain causality given the interconnectedness of these pathological events. For example, TNF-α and IL-1ß contribute to compromised BBB integrity [22], and the sequalae of BBB dysfunction, like invasion of peripheral immune cells, thus creating a positive feedback loop caused by the exponential increase in cytokine release from the invasive immune cells [30]. These signs of chronic inflammation have been demonstrated in post-mortem TBI tissue analysis through the presence of activated microglia, indicated by the expression of CD63 and CR3/43, for as long as 18 years post-injury [31,32].

Astrocytes, another neuroglial cells, are important for the regulation of extracellular glutamate in the brain and BBB permeability [3]. They are closely associated with the cerebral vasculature, where they participate in the vasoregulation of cerebral vasculature through the mediation of vasoactive compounds, such as NO, K^+^, adenosine, and arachidonic acid. As such, they comprise a key part of the ‘neurovascular unit’ described by Iadecola [33]. They may also serve a pro-inflammatory role similar to microglia and are even implicated in the dampening of acute neuroinflammation through the release of neurotrophins, anti-inflammatory cytokines, and prostaglandins [6]. After TBI, the chronic activation of astrocytes leads to astrocyte hypertrophy and the release of glial fibrillary acidic protein (GFAP), which contributes to neural scar formation and the inhibition of axon regeneration.

Chronic inflammation following TBI has also been implicated in the accumulation of hyperphosphorylated tubulin-associated unit (tau) proteins. Contrary to the previously assumed causative role of Aß plaques in the development of Alzheimer’s Disease (AD), it has been shown that cognitive decline in AD correlates with the accumulation of tau, and that Aß may even be a driver of downstream tau aggregation [34,35,36]. However, the pattern of pathologic tau protein aggregation in CTE, a neurodegenerative disease characterized by tau accumulation following TBI [37,38], differs significantly from what is observed in AD. In CTE, hyperphosphorylated tau accumulates in perivascular regions surrounding the original lesion, primarily in the superficial cortical layers, before spreading to other parts of the brain [39,40]. Depletion of microglia in mouse models with human tau isoform significantly slowed the deposition of tau, suggesting that microglia are involved in the propagation of hyperphosphorylated tau in the brain. The mechanism by which this occurs is thought to be microglial phagocytosis, and subsequent exocytosis, of tau. The same findings were not true of neurons or astrocytes [41].

### 2.2. Excitotoxicity and Neurotransmitter Dysregulation

Glutamate excitotoxicity is a phenomenon by which chronically elevated levels of glutamate, often due to impaired glutamate clearance mechanisms, cause mitochondrial dysfunction and eventually neuronal death [42,43]. Following TBI and BBB disruption, there is an acute increase in extracellular glutamate (as well as other neurotransmitters including aspartate, glycine, and GABA) which remains elevated [14,43,44]. Excess glutamate causes overactivation of the cation channels NMDA and AMPA, thus overloading neurons with calcium ions and precipitating cell death [42,45,46]. Evidence suggests that microglial activation also contributes to excitotoxicity by triggering NO release, which induces rapid glutamate release in mixed neuronal cultures with activated microglia and astrocytes [18]. Furthermore, NMDA receptor inhibition significantly reduced neuronal death, indicating that glial-induced excitotoxicity likely occurs through NO and NMDA receptor activation.

Astrocytes also have a role to play in TBI-induced excitotoxicity. Astrocytes normally clear the extracellular space of glutamate; however, their ability to do so is impaired post-TBI, leading to a potentially pathological increase in extracellular glutamate [3]. The aforementioned TNF-α and IL-1ß have been shown to alter the expression and distribution of glutamate receptors on astrocytes, further decreasing glutamate clearance following TBI.

Neuronal hyperexcitability has far-reaching consequences on greater neural circuits in addition to its local cytotoxic effects. Of these neural circuits, potentially the most affected are the layer V pyramidal neurons which are found in the cerebral cortex and amygdala. While not all morphologically similar between regions, they play an important role in the cortical synaptic input integration and have demonstrated involvement in higher cognitive function [47,48]. TBI disrupts the balance of inhibitory and excitatory input to layer V pyramidal neurons. In response, the brain attempts to remodel lesioned axons, but the newly formed synapses are predominantly excitatory. This disrupts the delicate balance between excitatory and inhibitory input and can predispose to epilepsy due to the heightened circuit activity in a phenomenon known as post-traumatic epilepsy (PTE) [48,49]. This has been demonstrated in PTE animal models which show increased glutamatergic signaling following TBI and a decreased number of inhibitory GABAergic synapses, particularly in layer V pyramidal neurons [50,51,52].

It is worth noting here that connections have been drawn between CTE and post-TBI epilepsy on the basis of shared miRNA epigenetic regulation, a topic discussed in a later section.

### 2.3. BBB Disruption

The BBB is a highly selective chemical and physical barrier responsible for monitoring and filtering blood as it enters CNS microvasculature [53,54]. Specialized endothelial cells, astrocytes, and pericytes line cerebral blood vessel walls with interlocking tight junctions and adherens junctions, creating a barrier unique to the CNS. The BBB is necessary in facilitating the uptake of nutrients and protecting the CNS from toxic and pathogenic substances [54]. Crucially, the BBB protects the brain from rapid fluctuations in electrolyte concentrations that are common in the periphery, thus safeguarding neuronal firing of action potentials in the CNS [55]. The shearing forces present during TBI can acutely disrupt the BBB, causing vasogenic edema with subsequent infiltration of peripheral immune cells, intraneuronal electrolyte dysregulation, and diffuse axonal injury due to disrupted microtubule architecture [6,14,15,53]. The circumventing of the BBB’s molecular sieve leads to the supraphysiologic concentration of complement cascade, substance P, and matrix metalloproteinase (MMP) activity [14,53,54,56,57,58].

Cerebral edema following TBI begins with peri-contusion swelling as ruptured cells release their contents into the interstitial space, increasing local osmolarity. Water then diffuses to the site of injury and causes swelling [59]. Secondary cytotoxic edema occurs as surrounding neurons upregulate aquaporin channels to absorb the water; however, the excessive influx can rupture the cells. When this phenomenon affects BBB cells, barrier compromise can cause vasogenic edema, further disrupting fluid homeostasis. The resulting elevation in ICP may compress cerebral blood vessels, leading to ischemic injury and, in severe cases, brainstem herniation and death [4,60].

The complement cascade is primarily involved in the identification, attack, and destruction of foreign pathogens, but it also serves several beneficial roles in the brain from synaptic pruning during development to cell cycle regulation. Though the complement is excluded from the brain by the BBB, the CNS has been shown to be capable of producing its own complement components, albeit at a far lower level than what is observed peripherally. The massive influx of complement and other peripheral immune agents during BBB disruption overwhelms brain tissue and rapidly induces the inflammation and death of neurons and astrocytes [56].

Substance P, a widely distributed neurotransmitter, interacts with the tachykinin receptor family to influence ion channel activity and gene expression. At high concentrations, it becomes immunogenic, contributing to cerebral edema and BBB disruption, as seen in post-mortem analyses of human brain tissue. MMPs, which regulate various cellular processes, further compromise the BBB by degrading its components. Studies on stroke and TBI show increased levels of MMP-2 and MMP-9 after injury, with MMP-2 reducing tight-junction integrity and MMP-9 causing complete degradation of the basal lamina and tight junctions [54,61,62].

As discussed previously, TBI can result in the deposition of hyperphosphorylated tau in perivascular spaces at the site of injury. A potential connection between chronic BBB disruption and CTE was shown by Blair et al., where perivascular tau deposition in Tg4510 mouse models coincided with the onset of BBB disruption, and inhibition of tau accumulation reduced the resulting BBB dysfunction [63].

### 2.4. Mitochondrial Dysfunction and Oxidative Stress

Mitochondria, membrane-bound organelles present in nearly all eukaryotic cells, primarily produce cellular energy in the form of ATP [64]. Following TBI, mitochondrial dysfunction is largely triggered by excitotoxicity and excessive calcium influx into neurons. Beyond promoting neuronal hyperexcitability, elevated calcium induces mitochondrial stress, leading to increased ROS production. Mouse models of TBI reveal that excess neuronal calcium correlates with mitochondrial swelling, membrane depolarization, reduced ATP production, and impaired calcium buffering capacity as early as 3 h post-injury. Electron microscopy further shows disrupted cristae, outer membrane swelling, and breakage, suggesting the involvement of the mitochondrial permeability transition pore (MPTP) [45,65]. The most notable effects of mitochondrial dysfunction are primarily mediated through decreased ATP production, increased ROS production, and mitochondrially driven apoptosis. As a general principle, much of a cell’s energy production is put toward maintaining ion gradients across the cell membrane, and neurons are no exception. When ATP production decreases, these ion gradients dissipate, which leads to severe cellular dysfunction and encourages apoptosis via MPTP [66]. This mechanism is supported by evidence showing that the closure of the MPTP with cyclosporine A mitigates oxidative stress and lessens neurodegeneration [67].

After TBI, ROS and reactive nitrogen species (RNS) contribute to neuronal and mitochondrial dysfunction through membrane lipid peroxidation. Evidence suggests that peroxynitrate, produced from the interaction of superoxide with NO, is one of the main radical species involved. Additionally, calcium-overloaded neurons are a major source of peroxynitrate, underscoring the role of excitotoxicity in contributing to mitochondrial stress-induced cell death [45,68]. There also exists a positive feedback loop between ROS and RNS production and Aß plaque deposition. Chronic microglial activation can lead to protein misfolding concurrent with the release of ROS and RNS, contributing to Aß deposition. The Aß plaques themselves elicit an inflammatory response from microglia and astrocytes, driving a positive feedback loop of chronic inflammation [3,69]. Mouse models of amyloid plaque show that inhibiting the Aß scavenger receptor on microglia, which reduces Aß clearance, increases TNF-α and IL-1ß levels. This evidence reinforces the physiological role of microglia in Aß clearance and that Aß contributes to chronic, microglia-driven inflammation [34,35,36]. Interestingly, in cases of CTE, tau protein may appear independently of Aß by the proposed mechanism of cis-tau production, which has been observed in human brain tissue and CSF following head injury. Mouse models of closed head injury treated with cis-tau antibody showed lessened cognitive decline and pathological development [70,71]. Taken together, the association between mitochondrial dysfunction and Aß and hyperphosphorylated tau deposition implicate mitochondrial dysfunction as a driver of neurodegenerative processes post-TBI.

Finally, oxidative stress has been linked with astrocytic glutamine synthase dysfunction, an enzyme which converts reclaimed, intracellular glutamate to glutamine for safe transport back to neurons. Decreased glutamine synthase activity causes increased levels of extracellular glutamate, which further exacerbates post-TBI hyperexcitability [3,72].

Oxidative stress is also theorized to play a role in the psychiatric sequelae of TBI, like major depressive disorder (MDD) and suicidality, as well as symptoms of behavioral dysregulation and short-term memory (STM) loss [73]. The mechanism of psychiatric symptoms after TBI may be in part due to the deposition of hyperphosphorylated tau and subsequent neurodegeneration in brain regions like the CA1 region of the hippocampus and amygdala, which are known to regulate memory and emotion, respectively [37,74]. Studies performed in MDD patients have demonstrated reduced levels of antioxidants with increased ROS level. Furthermore, depression is also linked to reduced ATP production in the brain, which itself is linked to reduced cognitive function and neuronal necrosis [66,75]. This mechanism of ROS-mediated MDD illustrates a clear link between TBI and psychiatric conditions like MDD and schizophrenia [76,77]. Overall, these data suggest a strong connection between post-TBI oxidative stress and adverse psychiatric outcomes.

### 2.5. Cytoskeletal Damage and Axonal Pathology

Microtubules are essential cytoskeletal proteins in neurons, providing the structural framework for transporting synaptic contents across the axon [78]. TBI damages microtubules through mechanical stress and several acute molecular disruptions. The initial mechanical injury causes diffuse axonal injury, marked by neuronal swelling followed by axonal degeneration due to microtubule breakdown at points of damage [79]. Molecular mechanisms contributing to microtubule disruption include IL-1β-mediated tau phosphorylation, which detaches tau from microtubules and may lead to its hyperphosphorylation [80]. Since IL-1β levels can remain elevated for over a decade post-injury, this offers a potential explanation for chronic microtubule dysfunction in TBI [81]. Additionally, elevated intracellular calcium after TBI activates calpain cysteine proteases, which cleave microtubules and exacerbate cellular dysfunction [53]. Inhibiting these proteases has been shown to reduce functional and behavioral deficits and attenuate cell death in animal models of TBI [82].

Tau is a protein that regulates microtubule assembly and spatial organization, thus playing a very important role in basic neuronal function [80,83,84]. The phosphorylation and dephosphorylation of tau are normal processes which regulate tau binding with microtubules. When tau is phosphorylated, its affinity for microtubules is reduced, causing it to disassociate [81,85]. The mechanical stress of TBI not only causes disruption and breakage of microtubules but also causes the dissociation of tau, eventually aggregating and becoming hyperphosphorylated [86]. The inhibition of tau pathology formation in drosophila models of tauopathies was associated with the reduced phosphorylation of monomeric tau protein, suggesting that the phosphorylation of tau not bound to microtubules is crucial for its aggregation [87]. Upon aggregation, tau proteins are observed to accumulate preferentially in the soma of the neuron [88]. Once hyperphosphorylated tau has seeded in neural tissue, it spreads throughout the brain, a phenomenon that has been famously investigated by Braak et al. [89]. The mechanism behind this dissemination is thought to be a combination of neuronal endocytosis of extracellular tau, since extracellular tau deposits are observed in CTE and AD even though tau is normally an intracellular protein exclusively, and neuron-to-neuron spread of tau aggregates in a manner similar to the spread of prions [90]. It remains unclear exactly how hyperphosphorylated tau transmitted between neurons induces further tau aggregation.

### 2.6. Epigenetic Modifications

TBI can lead to persistent changes in gene expression by altering these epigenetic mechanisms [91]. Some studies suggest that over 400 genes, related to both cell survival and death, experience DNA methylation post brain injury [92]. For example, brain IGF-1ß, a signaling molecule associated with neuronal growth, is upregulated following TBI, but phosphatases are downregulated in CTE, which negatively affects multiple pathways, including the MAPK and calcium signaling pathways, and may even contribute to tau hyperphosphorylation [92,93].

It should also be noted that animal models of TBI provide evidence for the epigenetic modification of writer and eraser genes facilitating the addition and removal of methyl groups from DNA, which suggests a potential mechanism by which TBI has such extensive effects on epigenetic regulation [91]. Micro RNA (miRNA), non-coding strands of RNA which target and silence mRNAs, dysregulation has been implicated in both TBI and post-TBI epileptogenesis [94]. A meta-analysis of this phenomenon found that 10 miRNAs are dysregulated in both TBI and epilepsy: (miR-27a, miR-502, miR-130b, miR-9, miR-625, miR-660, miR-138, miR-21, miR-30a and miR-1307). Of these miRNAs, six are associated with apoptosis (miRNA-27a, miRNA-130b, miRNA-9, miRNA-660, miRNA-21, and miRNA-138). Additionally, three of them are associated with arresting the cell cycle, inhibiting neurogenesis (miRNA-660, miRNA-130b, and miRNA-27a) [94]. These miRNAs may be involved in the pathogenesis of epilepsy after TBI.

It is well known from conditions like Rett syndrome, Angelman syndrome, Fragile X syndrome, and Huntington’s Disease that epigenetic dysregulation can have significant negative effects on cognition and physiology [95,96,97,98]. Though the nature of the epigenetic dysregulation following TBI may be different in origin from common genetic conditions caused by epigenetic dysregulation, they both nonetheless demonstrate that the epigenetic modulation of DNA can have devastating impacts on neurocognitive function.

### 2.7. Memory and Cognitive Dysfunction

Various animal studies have demonstrated both STM and long-term memory deficits following TBI, sometimes lasting for year [99,100]. Notably, TBI in rodent models has been shown to affect working memory, spatial anterograde and retrograde memory, and episodic memory [101,102,103,104,105,106]. The results of several of these studies indicate that the memory deficits persisted up to 3 months [107].

Memory and cognitive deficits following TBI have been well-documented, both pathologically and behaviorally, in humans [107,108,109]. After TBI, there is extensive neuronal damage and axonal injury to the hippocampus and the cerebellum [110]. The hippocampus is the region of the brain responsible for recall of memory, thus damage to the hippocampus is associated with memory deficits [107,108,111]. Specifically, TBI induces aberrant hyperexcitability in the hippocampal dentate gyrus, which is involved in pattern separation and cortical input filtering; hypoexcitability in the CA1 region, responsible for memory encoding and retrieval; and neuronal death in the CA3 region, which also contributes to memory processing [112,113,114,115,116]. This pattern aligns with a 2016 meta-analysis showing that individuals with TBI experience progressively worsening deficits in verbal STM, as well as verbal and visuospatial working memory [117]. Additionally, an analysis of over 15,000 people revealed that mild and moderate-to-severe TBI were associated with long-term impairments in attention and executive function [118].

### 2.8. Psychiatric Sequalae

TBI is associated with a number of psychiatric comorbidities, including post-traumatic stress disorder (PTSD) [119]. Individuals with TBI are at higher risk of developing PTSD, and a comorbid diagnosis is associated with poor clinical outcomes [119,120]. Rodent models subjected to closed-head blast injuries exhibit PTSD-like behaviors, have altered hippocampal circuits, and experienced oxidative stress [121,122,123]. These findings are particularly pertinent to the increasing incidence of blast-related TBI and subsequent PTSD in military veterans [124,125].

In humans, PTSD and TBI show similar fear response-enhancing patterns of white and gray matter abnormalities in the cortex and hippocampus. Neuroinflammation, excitotoxicity, and oxidative stress are common to both TBI and PTSD, highlighting a pathological link [124]. Furthermore, both conditions share a number of symptom clusters, including emotional dysregulation, enhanced fear learning, and fear generalization; these are underpinned by aberrant changes to neural circuits involved in emotional regulation and neural metabolism [119]. One aspect of this dysregulation is exemplified by the relationship between the hypothalamic–pituitary–adrenal axis, which promotes glutamate and glucocorticoid release in response to stress [119,126]. Both glutamate and glucocorticoid levels are elevated post-TBI, suggesting a shared molecular mechanism underlying post-traumatic stress and TBI-related cognitive deficits [43,119,127].

### 2.9. Preclinical Models of TBI

There are three common animal models used to study TBI: controlled cortical impact (CCI), fluid percussion injury (FPI), and weight drop/impact acceleration [128]. The FPI model was originally established in rabbits, but has since been adopted to murine models in the 1980s [129,130]. FPI is induced by rapidly transmitting a pulse through a fluid (i.e., saline) in direct contact with the exposed cranium, resulting in brain deformity due to increased ICP [128,131,132]. Both lateral (i.e., unilateral) and central (i.e., bilateral) FPI are well-validated and widely used methods of modeling TBI [129,131,133]. The CCI model was developed later in ferrets but quickly adapted for rodents [129,134,135]. Unlike FPI, CCI utilizes a piston driven at a specified speed and length into the exposed dura, allowing for precise control of TBI severity [128,129,134,136]. The weight drop/impact acceleration model is less popular than the FPI or CCI due to its lack of precision [128]. Similarly to the CCI method, it entails a piston positioned above the exposed cortex or cranium; however, instead of being electronically driven, the piston is propelled by the force of weight dropped from a fixed height [137,138].

It should be noted that these rodent models are used given their ease and affordability; however, their anatomical differences from human cranial and neural architecture may limit their clinical applicability [128,139]. These models have been adapted numerous times to study more specific types of head injuries, including closed-head TBI, repetitive TBI, blast TBI, penetrating head injury, and even shaken baby syndrome [138,140].

## 3. Diagnostic Approaches and Biomarkers

### 3.1. Molecular Biomarkers

Preclinical models had previously demonstrated that acute occurrences of tau aggregations were observed after TBI; therefore, tau was suggested to be a biomarker for TBI, as it is released into the bloodstream subsequently after trauma [141,142,143]. Clinical micro-dialysis in patients with significant TBI demonstrated that higher tau levels found within brain extracellular fluid were associated with worsening symptoms 6 months after injury [144]. Chronic implications of the tau biomarker are suggested, as studies have found that severe injuries or multiple TBIs lead to hyperphysiological tau accumulation in specific brain regions [145,146].

Astrocytes synthesize GFAP, a critical intermediate filament-associating protein involved in preserving the structural integrity of astrocytes [147,148]. If neurotrauma were to occur, GFAP is released into the CSF and bloodstream as a proxy of astrocytic and BBB damage [149]. GFAP serves as a reliable marker, as the protein is specific to the CNS and is not found in the bloodstream under physiological conditions. Common characteristics of acute neurotrauma include rapid elevation of GFAP levels after injury due to astrocyte death: this quick response allocated the FDA to approve blood tests to measure GFAP levels in TBI diagnoses. Chronic instances of neurotrauma have shown prolonged activation of astrocytes and accumulation of the GFAP, yielding clinical findings of continuous neuroinflammation and glial scarring [150].

Neurofilament Light Chain (NfL) is a variation of neurofilament that supports the neuronal cytoskeleton [151]. While NfL is primarily found within axons, particularly larger myelinated axons that require greater support, trauma can induce their release into the bloodstream. Unlike GFAP, NfL does not appear in the bloodstream until several days after injury since axonal neurofilaments demonstrate slow turnover, thus rendering it less reliable for acute diagnostics [152]. However, NfL can remain elevated in the bloodstream for up to 5 years after TBI, coinciding with white matter atrophy and cognitive decline [153,154].

Ubiquitin C-terminal Hydrolase L1 (UCH-L1) is an enzyme integral to the ubiquitin-proteosome system, as it recycles and removes ubiquitin markers from polyubiquitinated proteins [155]. UCH-L1 is predominantly conserved in neurons, thus serving as a reliable marker for neuronal damage. It is transiently increased in brain extracellular fluid within hours post-TBI; however, levels quickly return to baseline, thus rendering UCH-L1 a non-robust measure for chronic neurotrauma [156]. Despite this, UCH-L1 remains a promising clinical biomarker, as the FDA cleared UCH-L1 assays in blood tests [157].

S100 Calcium Binding Protein B (S100B) corresponds to intracellular calcium homeostasis and cell proliferation as well as neuroplasticity [158]. Raised levels of S100B in extracellular fluid correlate with damage to the BBB and astrocytes [159]. Furthermore, S100B levels have a proportional relationship with the severity of TBI, making it possible to assess the extent of intracranial lesions. In acute settings, S100B levels are quick to respond to injury and decay to physiological levels within 24 h for less severe injury [160]. Persistent neurotrauma can lead to continuously elevated levels of S100B, which has been implicated in the pathogenesis of CTE and AD [161].

### 3.2. Diagnostic Imaging

Diffusion Tensor Imaging (DTI) is an imaging technique that analyzes the anisotropy of water within brain tissue with the use of magnetic resonance imaging [162]. Typically, water molecules will diffuse parallel to axonal fibers, which can be measured via mean diffusivity and fractional anisotropy [163]. However, TBI can disrupt this movement through axonal injury and demyelination, leading to both increased mean diffusivity and decreased fractional anisotropy [164]. These differences can be quantified to assess the extent of neurotrauma within a patient and the degree of recovery over time. Moreover, advanced derivations of DTI can be utilized to monitor microstructural changes. The use of diffusion kurtosis imaging provides a more in-depth approach to imaging diffusion by measuring any variation in water diffusion based on non-Gaussian diffusion, allowing for a more realistic analysis [165]. Another derivative of DTI, high-definition fiber tracking (HDFT) aims to address complexities in fibers, including crossing and kissing fibers, by mapping entire tracts [166]. HDFT has been shown to capture subtle abnormalities in white matter tracts that would have been undetected with traditional DTI, although it is not yet as commonly utilized in the clinical setting [167].

Positron emission tomography (PET) utilizes gamma ray emitting tracers, such as 18F-flortaucipir and 18F-MK-6240, to quantify tau tangle deposition patterns [168,169]. This technique allows for the differentiation of tauopathies associated with TBI from those seen in other neurodegenerative diseases. Thus, PET scans can be integrated into longitudinal studies to monitor the progression of tau aggregation in chronic TBI and CTE [170,171]. PET can also image circumstances of neuroinflammation and glial activation in vivo via separate radiolabeled tracers, including 11C-PK11195, which bind to translocator proteins [172,173]. This subset of outer mitochondrial membrane proteins is upregulated in areas with inflammation. Alternative targets beyond translocator proteins are possible, including cyclooxygenase enzymes and cannabinoid receptors, both of which are upregulated in inflammation [174]. Newer tracers, such as 11C-DPA-713 and 18F-DPA-714, continue to be optimized for increased tissue, longer half-life, and lower non-specific binding.

Functional magnetic resonance imaging (fMRI) detects differences in oxygenated and deoxygenated blood flow, enabling spatial and temporal correlation between regional tissue oxygenation and brain activity [175]. This association can be used to identify instances of overcompensation and overactivation in areas like the posterior cingulate gyrus, especially when it fails to coordinate with other components of the default mode network (DMN) [176]. This altered activity is thought to reflect an inefficient use of neural resources post-TBI, as the brain attempts to maintain normal cognitive functions. Variants of fMRI offer added specificity; for instance, task-based fMRI assesses behavioral circuits related to decision making and motor coordination, while resting-state fMRI depicts changes within the DMN or salience network [177,178].

Magnetic resonance spectroscopy (MRS), a variant of traditional MRI, determines changes in metabolism that precede structural changes, allowing for earlier detection of neurotrauma [179]. MRS assessments utilize markers such as N-acetyl aspartate, which mark mitochondrial function. Decreases in N-acetyl aspartate imply neuronal loss or impaired metabolism implicit to TBI [180]. Likewise, increased lactate, a byproduct of anaerobic metabolism, detected by MRS, serves as proxy for mitochondrial dysfunction [181]. MRS allows for longitudinal assessment, enabling the monitoring of recovery or further degeneration in individual patients [182].

Imaging techniques to properly analyze the extent of BBB injury include dynamic contrast-enhanced MRI (DCE-MRI) and susceptibility-weighted imaging (SWI). DCE-MRI utilizes gadolinium-based contrast agents that are typically restricted by the BBB; however, disruption of the BBB can cause leakage of these agents into the brain parenchyma. The extent of leakage can be quantified and used to assess TBI severity [62,183]. SWI is based on the magnetic properties of tissues that arise from differing amounts of paramagnetic substances, such as iron or deoxyhemoglobin, present in blood vessels [184]. Microhemorrhages post-TBI can be detected by the presence of these substances, reflecting the extent of mechanical brain damage [185].

Long-term monitoring of post-TBI pathology can help identify optimal windows for targeted therapies and biomarker analysis, enhancing the accuracy of injury assessment and its progression over time. Molecular biomarkers, like tau, GFAP, NfL, UCH-L1, and S100B, determine structural neuronal damage and white matter degradation while inflammatory markers, including cytokines and chemokines, provide insight on the state of injury [186]. Furthermore, alterations in metabolism suggesting mitochondrial dysfunction can be assessed via N-acetyl aspartate and lactate [187]. Biomarker data has been successfully used to identify patients in need of advanced imaging for targeted follow-up [188]. Combinations of biomarkers with imaging techniques can underscore causal relationships and provide datasets for predictive modeling, as shown in studies that successfully used machine learning to combine biomarker and imaging data to predict patient outlook up to 6 months after injury [189].

## 4. Therapeutic Strategies and Future Directions

To mitigate or even reverse secondary brain injury following TBI, several clinical and therapeutic strategies have been designed by targeting the pathways that have been shown to play a crucial role in the molecular mechanisms that lead to injury (Table 1).

The principal method of mitigating secondary brain injury is through diligent clinical management of TBIs to lessen the likelihood of further deterioration. These preventative measures begin even before reaching the hospital, with on-site emergency treatment of TBI via stabilization of blood pressure, immobilization of the spine, and rapid intubation being associated with lower mortality rates [190]. Upon arriving at the hospital, the primary focus of the healthcare team is to monitor ICP, as chronic elevation, even days after injury, can cause increased leukocytosis and the release of pro-inflammatory cytokines and chemokines which drive the inflammatory changes that lead to secondary brain injury [191,192]. Therefore, the management of increased ICP through fluid balance, optimized blood osmolarity, seizure mitigation, ketamine, or decompressive surgery is critical in the treatment of the primary TBI to prevent secondary brain injury [192,193,194,195,196]. Further studies have also examined the role that nutritional supplementation can play in mitigating secondary brain injury, with creatine, omega-3s, and antioxidants showing a protective effect [197].

Despite the best efforts to mitigate secondary brain injury through neuroprotective measures, damage can still arise, which necessitates a robust arsenal of pharmaceutics, biomaterials, and other therapeutic interventions. Unfortunately, there are currently no definitive FDA-approved disease modifying therapies for secondary brain injuries which arise following TBI [197]. Therefore, considerable effort has been dedicated to investigating new treatments repurposing FDA-approved medications for other indications. A promising pharmaceutical approach has been to target the inflammatory pathways present in secondary brain injury, specifically by modulating microglial activation and associated signaling cascades [6]. Therapeutic agents in this space aim to shift microglial activity toward a neuroprotective state by targeting inflammatory pathways such as TLR4/NF-κB, MAPK, and JAK/STAT [198]. For example, adenosine A3 receptor agonists have been shown to attenuate neuroinflammation by reducing NF-κB and MAPK pathway activation, leading to decreased downstream NOD-like inflammasome activation [199]. Additionally, preclinical trials with minocycline, a tetracycline antibiotic, have suppressed TLR4/NF-κB pathway activation, reducing pro-inflammatory cytokine release and improving functional recovery after TBI [200]. Many other anti-inflammatory agents have been investigated with mixed results for secondary brain injury, including immunomodulators such as anakinra and natalizumab, inflammatory mediators such as VX-765 (a caspase-1 inhibitor), statins, and corticosteroids [201,202,203,204,205]. While promising, there is still a demand for robust outcomes in Phase III and Phase IV clinical trials for anti-inflammatory therapeutics. For instance, recent Phase III clinical trials investigating novel anti-inflammatory and neuroprotective agents like ronopterin (VAS-203), a promising nitric oxide synthase inhibitor, have failed to show significant results [206].

Other approaches which aim to treat secondary brain injury focus on modifying gene expression to reduce neuroinflammation and enhance neuroprotection via epigenetics [207]. Histone deacetylase (HDAC) inhibitors, such as vorinostat, have shown promise in preclinical models by promoting neuronal survival and reducing pro-inflammatory cytokines [208]. Additionally, targeting microRNAs such as miR-21 can modulate immune responses and apoptosis pathways, offering a novel therapeutic strategy for mitigating secondary brain injury [209].

Even more promising are strategies designed to reverse damage and regenerate lost structures using stem cells and biomaterials. Pre-clinical studies of stem cell therapies, particularly those involving mesenchymal stem cells (MSCs) and neuro progenitor cells (NPCs) have demonstrated potential in restoring neuronal function after TBI by migrating to damaged brain tissue, differentiating into neural and glial cells, and secreting anti-inflammatory factors and growth factors that promote repair [210]. For example, a study of transplanted MSCs in a mouse model of TBI showed decreased serum levels of inflammatory cytokines, such as TNF-α and IL-1β, and stimulated adult NPC proliferation [211]. Other promising regenerative strategies involve the use of biomaterials designed to activate cellular repair mechanisms and promote axonal repair in TBI models [212]. Other therapeutics use biomaterial-based drug delivery for the controlled and targeted release of anti-inflammatory agents to sites of injury [213].

Ultimately, further research is needed to better understand TBI progression to improve current experimental therapies, develop more effective treatments, and possibly offer diagnostic and prognostic clinical testing. Integrating fluid biomarkers with advanced imaging techniques could offer a comprehensive approach to understanding these mechanisms [214]. Studies have demonstrated that blood-based biomarkers, such as GFAP and UCH-L1, can provide insights into brain injury and assess the severity of TBI when paired with MRI [215]. When combined with blood biomarkers, this advanced MRI imaging approach provides a more precise understanding of TBI and progression [216]. However, like many TBI-related techniques, it still faces fundamental challenges, and further research is needed to develop standardized methods before it can be widely implemented in clinical practice [214]. Thus, despite the promise of therapies discussed here, further pre-clinical and long-term clinical trials are needed to thoroughly evaluate the efficacy of interventions aimed at mitigating chronic neurodegeneration.

**Table 1:** A selection of ongoing clinical trials of biomarker utility as of 6 June 2025. Enrollment totals are actual unless stated otherwise. GCS = Glasgow Coma Scale. Information was gathered from the National Library of Medicine, Clinical Trials.gov Registry [217].

Biomarker	Inclusion Criteria	Recruitment Status	Enrollment	Sponsor	Clinical Trial Identifier
GFAP	>18 years old, neurological symptoms	Unknown Status	120	Hospices Civils de Lyon	NCT05742087
GFAP/UCH-L1	>18 years old, GCS 13–15	Recruiting	1500 (estimated)	Centre Hospitalier Princesse Grace	NCT05885529
S100B/GFAP	18–100 years old, GCS 3–8	Completed	6	University of Aarhus	NCT03062566
S100B/GFAP	>18 years old, GCS 13–15	Completed	595	University of Aarhus	NCT02867137
GFAP/UCH-L1	>18 years old, GCS 13–15	Completed	194	Banyan Biomarkers, Inc.	NCT02541123
GFAP/UCH-L1	0–20 years old, GCS <12	Completed	77	Valleywise Health	NCT02609568
S100B	>18 years old, GCS 15	Completed	1025	University Hospital, Clermont-Ferrand	NCT04543162
GFAP/UCH-L1	>18 years old, GCS 13–15	Completed	1501	University Hospital, Grenoble	NCT04032509
GFAP/UCH-L1	>18 years old, GCS 9–15	Completed	119	Banyan Biomarkers, Inc.	NCT02439736

## 5. Conclusions

TBI is not a singular event but the beginning of a complex cascade of molecular and cellular disruptions that can lead to long-term neurodegeneration. The mechanisms of secondary injury, including neuroinflammation, mitochondrial dysfunction, and BBB disruption, create a progressive cycle that exacerbates neuronal dysfunction and increases the risk for chronic conditions such as AD and CTE [1]. Persistent inflammation, metabolic failure, and excitotoxic damage contribute to prolonged neurological impairment, highlighting the need for early detection and targeted intervention [2].

Recent advances in biomarkers (e.g., tau, GFAP, Nfl, UCH-L1) and neuroimaging modalities (e.g., DTI, MRS) have enhanced diagnostic accuracy and enabled differentiation between acute and chronic phases of injury [179,192]. Despite these advancements, there remains a lack of FDA-approved therapies specifically designed to mitigate secondary brain injury [179]. Experimental treatments, including microglia-modulating drugs, HDAC inhibitors, and stem cell therapies, show promise but lack sufficient clinical validation [148].

Beyond biological complexity, TBI also presents a major public health challenge, with disproportionate impacts on racial and ethnic minorities, military personnel, and individuals in rural communities. Integrating precision diagnostics with neuroprotective strategies, we can shift the focus from acute survival to long-term neuroprotection, improving outcomes for individuals affected by TBI.

## Abbreviations

The following abbreviations are used in this manuscript:

TBI	Traumatic Brain Injury
CTE	Chronic Traumatic Encephalopathy
GFAP	Glial Fibrillary Acidic Protein
NfL	Neurofilament Light Chain
UCH-L1	Ubiquitin C-terminal Hydrolase L1
BBB	Blood brain barrier
ICP	Intracranial pressure
DAMP	Damage-associated molecular pathogen
ROS	Reactive oxygen species
FADD	Fas-associated death domain
Aß	Amyloid beta
tau	Tubulin-associated unit
AD	Alzheimer’s Disease
PTE	Post-traumatic epilepsy
MMP	Matrix Metalloproteinase
MPTP	Mitochondrial Permeability Transition Pore
RNS	Reactive nitrogen species
MDD	Major Depressive Disorder
STM	Short-term memory
miRNA	Micro RNA
PTSD	Post-traumatic stress disorder
CCI	Controlled cortical impact model
FPI	Fluid percussion injury model
S100B	S100 Calcium Binding Protein
DTI	Diffusion Tensor Imaging
HDFT	High-definition fiber tracking
PET	Positron Emission Tomography
fMRI	Functional magnetic resonance imaging
DMN	Default mode network
MRS	Magnetic Resonance Imaging
DCE-MRI	Dynamic contrast-enhanced MRI
SWI	Susceptibility-weighted imaging
HDAC	Histone deacetylase
MSC	Mesenchymal stem cell
NPC	Neuroprogenitor cell
